# Genome-wide analysis of the ALDH superfamily in *Castanea mollissima* highlights roles in abiotic stress responses

**DOI:** 10.3389/fpls.2026.1808001

**Published:** 2026-06-02

**Authors:** Xiangyu Wang, Tong Zhang, Yujuan Tian, Jinxin Wang, Xili Liu, Guoyun Zhang, Dongsheng Wang, Haie Zhang, Liyang Yu

**Affiliations:** 1 The Office of Scientific Research, Hebei Normal University of Science and Technology, Qinhuangdao, Hebei, China; 2 Powerchina Northwest Engineering Corporation Limited, Xian, Shaanxi, China; 3 Engineering Research Center of Chestnut Industry Technology, Ministry of Education, Hebei Normal University of Science and Technology, Qinhuangdao, Hebei, China; 4 Shijiazhuang Institute of Pomology, Hebei Academy of Agriculture and Forestry Sciences, Shijiazhuang, Hebei, China; 5 Research Institute of Forestry, Chinese Academy of Forestry Sciences, Beijing, China; 6 Hebei Key Laboratory of Horticultural Germplasm Excavation and Innovative Utilization, College of Horticultural Science and Technology, Hebei Normal University of Science and Technology, Changli, Hebei, China

**Keywords:** abiotic stress, aldehyde dehydrogenase superfamily, *Castanea mollissima*, expression analysis, phylogenetic evolution

## Abstract

Abiotic stresses significantly impede the growth and yield of plants. In this context, the aldehyde dehydrogenase (ALDH) superfamily is integral to plant stress responses. To date, the systematic identification and functional characterization of the *Castanea mollissima* ALDH superfamily have not been reported. This study represents the first genome-wide systematic analysis of the CmALDH superfamily, identifying 25 *CmALDH* genes across nine families. Phylogenetic analysis suggests that members of the CmALDH superfamily underwent purifying selection during evolution, predominantly driven by dispersed duplication events. Collinearity analysis demonstrated both conservation and species-specific variations of *ALDH* genes between *C. mollissima* and several other species. Examination of *cis*-acting elements revealed that *CmALDH* promoter regions contain numerous elements associated with hormone response, abiotic stress, and light response. Transcriptome data analysis for six abiotic stresses (high temperature, low temperature, drought, salt, alkalinity, and shading) and RT−qPCR validation (high temperature, low temperature, salt, and alkaline stresses) revealed differential expression of various *CmALDH* genes, suggesting their potential roles in stress responses. This study systematically elucidates the evolutionary characteristics and abiotic stress response mechanisms of the CmALDH superfamily, providing valuable genetic resources and a theoretical basis for further analysis of the molecular underpinnings of stress resistance in woody plants and for the genetic enhancement of resistance in *C. mollissima*.

## Introduction

Abiotic stresses, such as drought, salinity, extreme temperatures, and heavy metal contamination, pose significant challenges to crop growth, development, and geographic distribution, severely threatening global agricultural productivity and ecological stability (Gong et al., 2020; Jiang et al., 2025). Over the course of evolution, plants have developed intricate physiological, biochemical, and molecular mechanisms to counteract these stresses (Missihoun et al., 2018). These adaptive responses include the synthesis of osmoprotectants, activation of reactive oxygen species (ROS) scavenging systems, and the regulation of stress-responsive gene expression (Huang et al., 2012). Among the metabolic byproducts induced by stress, aldehydes accumulate excessively, leading to cytotoxicity and disruption of cellular structures, including proteins and nucleic acids. Consequently, efficient clearance of aldehydes is imperative for maintaining cellular homeostasis (Kotchoni et al., 2006). The aldehyde dehydrogenase (ALDH) superfamily is critical for the detoxification of aldehydes (Islam and Ghosh, 2022; Su et al., 2022).

ALDHs are enzymes that utilize NAD+/NADP+ as cofactors and catalyze the irreversible oxidation of both endogenous and exogenous aromatic and aliphatic aldehydes into their corresponding carboxylic acids. This enzymatic activity not only mitigates the toxic effects of aldehydes but also aids plants in adapting to environmental changes by maintaining aldehyde homeostasis (Kirch et al., 2004; Tola et al., 2020; Islam et al., 2022). The ALDH superfamily is classified into 24 families (ALDH1-ALDH24) based on sequence similarity and is widely distributed across different organisms (Black and Vasiliou, 2009). In plants, at least 14 ALDH families have been identified, seven of which are plant-specific, including ALDH11, 12, 19, 21, 22, 23, and 24. Notably, the ALDH19 family has been reported only in *Solanum lycopersicum*, while the ALDH23 and ALDH24 families are exclusive to *Selaginella moellendorfii*, *Physcomitrella patens*, and *Chlamydomonas reinhardtii* (Wood, 2009; Brocker et al., 2013; Jimenez-Lopez et al., 2016; Islam and Ghosh, 2022). Most proteins encoded by *ALDH* genes contain three conserved domains: 1) the catalytic thiol site (PS00070); 2) the glutamic acid active site (PS00687); and 3) the Rossmann fold (GxGxxG) coenzyme binding site (Islam and Ghosh, 2022).

The functions of *ALDH* genes in plant growth and development, as well as their roles in responses to abiotic stresses, have been extensively studied. For instance, *ALDH3F1* in *Arabidopsis thaliana* encodes an ALDH that modulates the flowering time by regulating *H3K9Ac* at the FLC locus; mutations in this gene lead to premature flowering, whereas its overexpression results in delayed flowering (Xu et al., 2020). In *Oryza sativa*, the *OsALDH2b* gene is crucial for normal anther development; mutants exhibit premature tapetum degeneration and abnormal microspore development (Xie et al., 2020). *PusALDH1* enhances the accumulation of volatile aromatics in *Pyrus ussuriensis* fruit (Chen et al., 2023). The expression of *ScALDH21* in transgenic *Gossypium hirsutum* enhances drought tolerance by increasing ROS scavenging capacity, alleviating osmotic stress (Yang et al., 2016). Overexpression of *ZmALDH* in *A. thaliana* enhances aluminum tolerance through strengthening the ascorbate-glutathione cycle, increasing the activity of antioxidant enzymes and related gene expression, reducing malondialdehyde accumulation, and enhancing proline synthesis (Du et al., 2022). *OsALDH7* plays a pivotal role in maintaining the viability of *O. sativa* seeds by regulating the levels of aldehydes produced during seed drying (Shin et al., 2009). Given the significance of *ALDH* genes, genome-wide analyses have been completed for various plants, including *A. thaliana* (14 genes) (Kirch et al., 2004), *O. sativa* (20 genes) (Gao and Han, 2009), *S. lycopersicum* (29 genes) (Jimenez-Lopez et al., 2016), *Zea mays* (24 genes) (Jimenez-Lopez et al., 2010), *Ziziphus jujuba* (19 genes) (Li et al., 2023), *Phyllostachys edulis* (60 genes) (Xu et al., 2023), *Arachis hypogaea* (71 genes) (Zhang et al., 2023), *Capsicum annuum* (28 genes) (Yang et al., 2025), and *Phaseolus vulgaris* (26 genes) (Eren, 2024). However, the evolutionary and functional characteristics of the ALDH superfamily in *Castanea mollissima* have not been investigated.

The genus *Castanea* is an important woody plant within the family Fagaceae, widely distributed across temperate regions of the Northern Hemisphere (Hu et al., 2022; Li et al., 2022). *C. mollissima*, the primary cultivated species, plays a significant role in mountainous agricultural and forestry economies (Yang et al., 2018; Hu et al., 2022). However, *C. mollissima* is frequently exposed to seasonal extreme temperatures, drought, and soil salinization/alkalization in its main cultivation regions, which severely affect its growth and productivity (Wang et al., 2024; Yu et al., 2025a, 2025). Despite numerous studies on the genetic breeding and physiological resistance of *C. mollissima*, systematic investigations into its molecular stress response mechanisms, particularly those involving key enzyme gene families like ALDH, are still lacking. Given that *ALDH* genes are known to participate in detoxification processes under various abiotic stresses, understanding their expression patterns under these conditions is crucial for elucidating their roles in *C. mollissima* stress adaptation. Therefore, in this study, we examined the expression of *CmALDH* genes under high temperature, low temperature, salt, alkali, drought, and shading stresses to systematically evaluate their potential involvement in the major environmental constraints faced by *C. mollissima*.

This study aims to conduct a comprehensive genome-wide identification, classification, evolutionary analysis, and expression profiling of the *C. mollissima* ALDH superfamily, focusing on their response patterns under abiotic stress. The specific objectives of this research are to systematically identify *ALDH* superfamily members in the *C. mollissima* genome, analyze their chromosomal distribution, gene structure, conserved motifs, and the physicochemical properties of their encoded proteins. The phylogenetic analysis will clarify the evolutionary relationships among *ALDH* members in *C. mollissima* and model plants. Collinearity analysis will explore the duplication patterns of *CmALDH* genes and their collinear relationships across multiple species. Expression dynamics of *CmALDH* genes under abiotic stresses such as extreme temperatures, high salinity, high alkalinity, and drought will be examined through transcriptomic data and RT-qPCR experiments. These findings are expected to enrich our understanding of the ALDH superfamily in woody plants, particularly the potential role of *CmALDH* genes in *C. mollissima* adaptation to abiotic stress and provide candidate genes and theoretical basis for improving *C. mollissima* stress resistance through genetic engineering.

## Materials and methods

### Identification of *ALDH* genes in *C. mollissima*

The genome sequence, protein sequences, and genome annotation files of *C. mollissima* were downloaded from the Castanea Genome Database (http://castaneadb.net/#/). Information on ALDH superfamily members in *A. thaliana* was obtained from previous studies, with their protein sequences downloaded from the PlantTFDB database (https://planttfdb.gao-lab.org/) (Islam et al., 2022). The Hidden Markov Model (HMM) for the ALDH domain (PF00171) was obtained from the Pfam database (http://pfam-legacy.xfam.org/). HMMER 3.0 software was utilized to search the *C. mollissima* protein files for sequences containing the PF00171 domain. Concurrently, BLASTP searches were conducted on all *C. mollissima* protein sequences using the ALDH protein sequences from *A. thaliana* as the reference sequence (Lee et al., 2014). Candidate sequences identified through both methods were submitted to NCBI-CDD for further validation to confirm the presence of PF00171 domain.

### Sequence analysis

The Protein Parameter Calculator function in TBtools was employed to determine the CmALDH protein’s physicochemical characteristics, encompassing isoelectric point, molecular weight, as well as amino acid length (Chen et al., 2020). Using the GFF3 file of the *C. mollissima* genome, TBtools software generated the exon-intron structure diagrams for *CmALDH* genes (Chen et al., 2020). We used the MEME online tool with default settings to identify conserved motifs in the *CmALDH* proteins. The resulting data file from NCBI-CDD, which contains information on conserved domains, was analyzed. Predictions of subcellular localization for *CmALDH* members were performed using the online tool Cell-Ploc (Chou and Shen, 2010). We also predicted the secondary structures of CmALDH proteins using SOPMA (Geourjon and Deléage, 1995) and their three-dimensional structures with SWISS-MODEL.

### Phylogenetic and *cis*-acting elements analysis

The *ALDH* genes from *A. thaliana*, *O. sativa*, *Sorghum bicolor*, *Vitis vinifera*, and *Z. mays* were retrieved from previous studies, and their protein sequences were downloaded from the PlantTFDB database (Islam et al., 2022). We performed a multiple sequence alignment of the amino acid sequences of these ALDH proteins using ClustalW (Kumar et al., 2016). Subsequently, a phylogenetic tree was constructed employing MEGA 7.0 software through a maximum likelihood analysis with 1000 bootstrap iterations (Kumar et al., 2016). The phylogenetic tree was visualized and refined using ITOL. Additionally, the sequence 2000 bp upstream of the start codon of the *CmALDH* genes was obtained using TBtools and analyzed for *cis*-acting elements using the PlantCARE database (Chen et al., 2020). The results were compiled and summarized. We used TBtools for quantifying and visualizing elements’ types and numbers (Chen et al., 2020).

### Chromosomal distribution and collinear analysis

Chromosomal location data for the *CmALDH* genes were extracted from the GFF3 file of the *C. mollissima* genome and visualized using TBtools (Chen et al., 2020). Collinearity within the *C. mollissima* genome was analyzed using MCScanX, and the type of duplication of the *CmALDH* genes was determined using the duplicate_gene_classifier tool (Wang et al., 2012). We differentiated between whole-genome duplication (WGD) and segmental duplication by analyzing the synonymous substitution sites (Ks) values of homologous genes within collinearity blocks, following the methodology described in a previous study (Yu et al., 2025b). The non-synonymous (Ka) and Ks values for gene pairs were calculated using the add_ka_and_ks_to_collinearity tool within MCScanX. The median Ks value for homologous gene pairs within collinearity blocks was computed using a custom script (Yu et al., 2025b). Genome data for *A. thaliana*, *Quercus robur*, *V. vinifera*, *O. sativa*, and *S. lycopersicum* were obtained from the Phytozome database (https://phytozome-next.jgi.doe.gov/). MCScanX was also employed to analyze collinearity between *C. mollissima* and these plant genomes.

### Functional enrichment and interaction network analysis

Functional annotation information for *C. mollissima* genes was sourced using eggNOG-mapper (Cantalapiedra et al., 2021). GO and KEGG functional enrichment analyses, along with visualization, were conducted using TBtools (Chen et al., 2020). The 2000 bp upstream sequence of the *CmALDH* promoters was submitted to the Plant Transcriptional Regulatory Map database (https://plantregmap.gao-lab.org/) to predict potential transcription factors (TFs) acting in this region. The STRING database was utilized to predict the interaction network of the CmALDH protein. Visualization of the predicted TF interaction network and protein interaction network was accomplished using Cytoscape (v3.9.1) (Kohl et al., 2011).

### Transcriptome analysis

RNA-seq data for *C. mollissima* under conditions of high temperature, low temperature, drought, salt, and alkaline stress were obtained from the NCBI SRA database, while data for shading stress were sourced from the NGDC database. These datasets were derived from five projects, identified by the following accession numbers: PRJNA1166987 (high and low temperature stress), PRJNA731244 (drought stress), PRJNA1363574 (salt stress), PRJNA1384844 (alkaline stress), and CRA022911 (shade stress). The stress treatments applied in these projects were as follows: For high−temperature stress, plants were exposed to 45 °C for 0, 4, 8, and 12 h; for low−temperature stress, plants were treated at −15 °C for 0, 5, 10, and 15 h; for salt stress, a 200 mmol/L NaCl solution was applied for 14 and 26 days; for alkaline stress, 0.00 g/L (control), 0.02 g/L and 0.5 g/L Na_2_CO_3_ solutions were applied for 7 days; for drought stress, water was withheld for 0, 10, 20, 30, and 40 days; for shading stress, plants were grown under 0 %, 50 %, 75 %, and 95 % shade. For the expression profiling analysis, RNA-seq expression levels (FPKM) were log2-transformed. Heatmaps illustrating the expression profiles were generated using TBtools (Chen et al., 2020).

### RT−qPCR analysis

The experimental material comprised 60-day-old seedlings of the ‘Yanshanzaofeng’ variety of *C. mollissima*, cultivated at Hebei Normal University of Science and Technology. The low-temperature treatment involved maintaining plants at -15 °C for 0, 5, 10, and 15 hours, respectively. Conversely, high-temperature stress entailed exposing plants to 45 °C for 0, 4, 8, and 12 hours. Salt stress treatments included the application of a 200 mmol/L NaCl solution, with distilled water serving as the control, over durations of 14, and 26 days. For alkaline stress, treatments with 0.00 g/L (control), 0.02 g/L and 0.5 g/L Na_2_CO_3_ solutions were administered, along with distilled water as a control, for a period of seven days. The experiment was arranged in a completely randomized design with three biological replicates per treatment, and each biological replicate consisted of leaf samples pooled from three individual seedlings. Leaf samples were promptly harvested following each treatment, immediately frozen in liquid nitrogen, and stored at -80 °C. Total RNA was extracted from the leaves of *C. mollissima* using an RNA extraction kit (Tiangen, Beijing). RNA integrity and concentration were assessed using a capillary electrophoresis system (Caliper), and only RNA samples with high integrity were used for subsequent analysis. The electropherograms are provided in **Supplementary Figure 1**. Subsequently, RNA was reverse-transcribed into cDNA using a reverse transcription kit (Takara, Dalian). Quantitative PCR analysis was conducted using the TB Green Premix Ex Taq Reagent Kit (Takara, Dalian) on an ABI 7500 real-time fluorescent quantitative PCR system (ABI, USA), employing the *actin* gene as the internal reference. Specific primers were used as detailed in **Supplementary Table 1**. All qPCR reactions were performed in three technical replicates. The relative expression levels of the *CmALDH* genes under the aforementioned treatments were quantified using the 2^-ΔΔCt^ method. One-way analysis of variance (ANOVA) followed by Tukey’s *post-hoc* test was applied to determine significant differences among treatment groups, with a significance level set at p < 0.05. A schematic overview of the experimental design, including stress treatments, sampling time points, and the number of biological replicates, is provided in **Supplementary Figure 2**. Data visualization and statistical analysis of significant differences were performed using GraphPad Prism 9.5 software (Livak and Schmittgen, 2001). In this study, expression analysis was performed using leaf tissue to ensure consistency across multiple stress treatments. However, given the important roles of roots in sensing drought and salt stress, future investigations should include root tissues to provide a more comprehensive understanding of *CmALDH* genes responses under these conditions.

## Results

### Identification, phylogenetic, and physicochemical properties analysis

**Figure 1:**
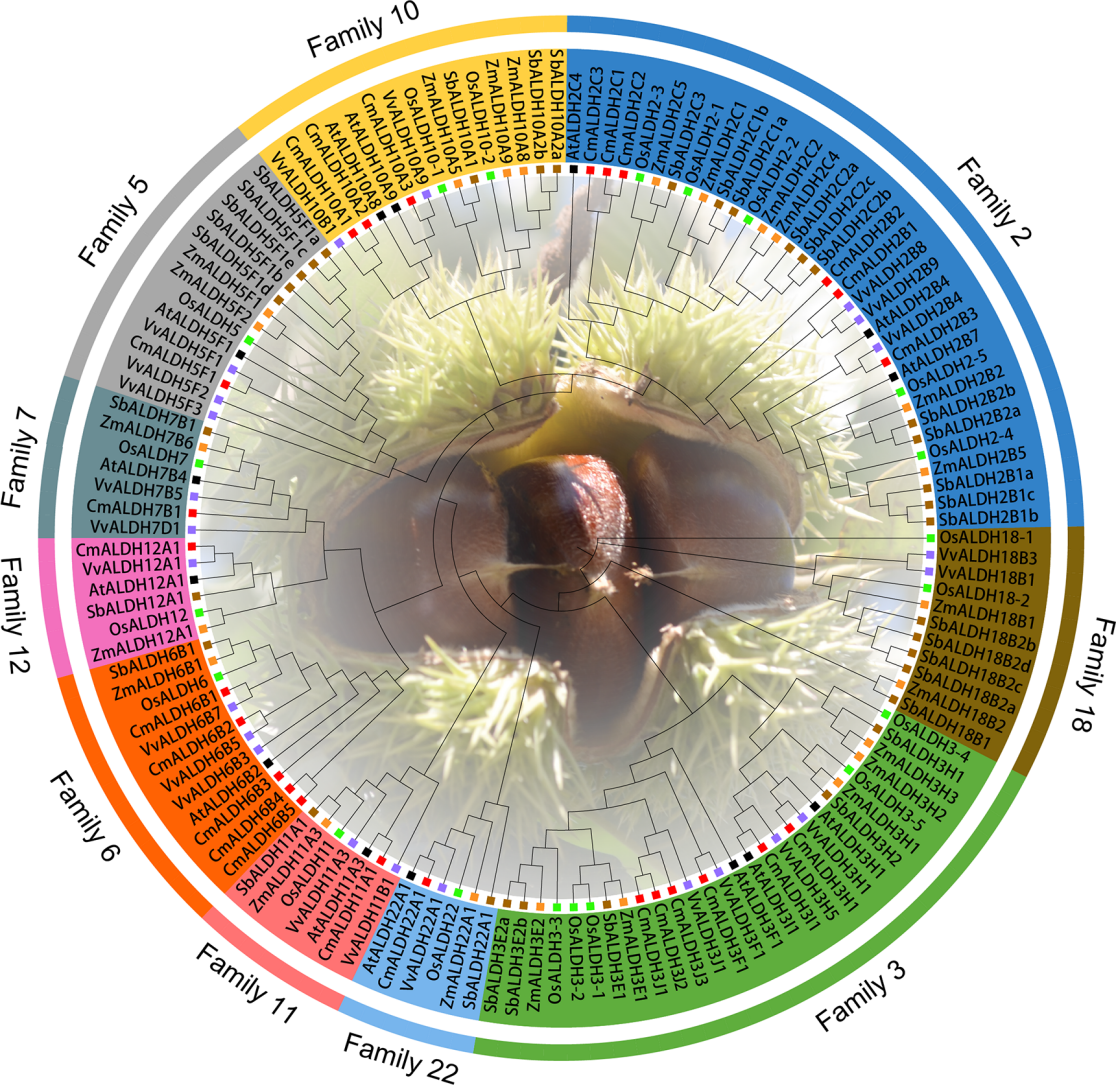
The phylogenetic tree of 139 ALDH proteins from *C. mollissima* (25), *A. thaliana* (14), *O. sativa* (20), *S. bicolor* (34), *V. vinifera* (23), *Z. mays* (23) was divided into ten families: family 2, 3, 5, 6, 7, 10, 11, 12, 18, and 22. The ten arcs in different colors on the outer circle represent ten families, while the six squares in different colors on the inner circle represent ALDH members from six plant species (*C. mollissima*, *A. thaliana*, *O. sativa*, *S. bicolor*, *V. vinifera*, *Z. mays*). MEGA 7.0 was used to construct the phylogenetic tree based on the protein sequences with the maximum likelihood method.

We identified 25 *CmALDH* genes in the *C. mollissima* genome (**Supplementary Table 2**). Phylogenetic analysis, utilizing ALDH protein sequences from *C. mollissima*, *A. thaliana*, *O. sativa*, *S. bicolor*, *V. vinifera*, and *Z. mays*, classified 139 members into 10 distinct families. The distribution of *CmALDHs* across these families varied significantly (**Figure 1**). Families 2 and 3 comprised the largest groups, each containing six *CmALDH* members, whereas families 5, 7, 11, 12, and 22 each included only one member. Notably, family 18 contained no *CmALDH* members. The proteins encoded by these genes varied in length from 124 to 1065 amino acid residues, with molecular weights ranging approximately from 13.56 kDa to 116.42 kDa. The theoretical isoelectric points of the CmALDH proteins ranged from 4.68 to 8.81 (**Supplementary Table 2**). A majority of the CmALDH proteins (18 out of 25) exhibited instability indices below 40, suggesting potential stability. The average hydrophilicity values of these proteins spanned from -0.401 to 0.229. To characterize the structural aspects, both secondary and tertiary structures of the CmALDH proteins were predicted. The analysis indicated that the secondary structure predominantly consisted of random coils (ranging from 39.71% to 57.75%), followed by alpha helices (29.03% to 44.64%) and extended strands (10.7% to 17.55%), with a complete absence of beta-turns (**Supplementary Table 3**). Tertiary structure predictions suggested that proteins within the same subfamily exhibited similar conformations (**Supplementary Figure 3**). Predictions of subcellular localization indicated that most CmALDH proteins are primarily located in the cytoplasm (9 out of 25) and chloroplasts (6 out of 25), with others potentially distributed in the nucleus, vacuole, cell membrane, and various other locations (**Supplementary Table 2**).

**Figure 2:**
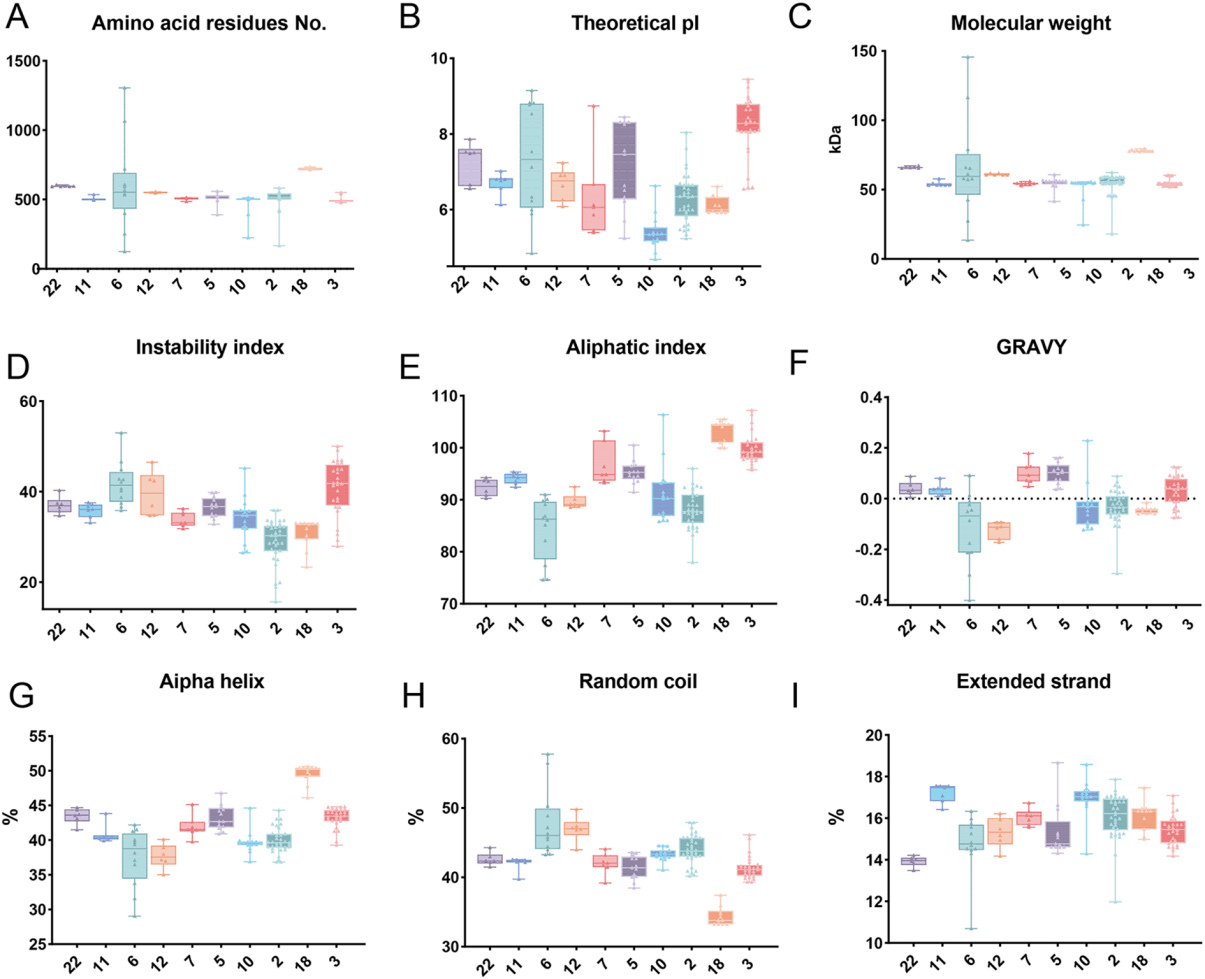
The physicochemical properties and two-dimensional structure prediction of members of the ALDH family (from six plant species: *C. mollissima*, *A. thaliana*, *O. sativa*, *S. bicolor*, *V. vinifera*, *Z. mays*) among ten families. **(A-I)** The number of amino acid residue, theoretical pI, molecular weight, instability index, aliphatic index, grand average of hydropathicity (GRAVY), as well as the proportions of alpha helix, random coil, and extended strand for ten families of the ALDH superfamily.

Additionally, we analyzed the physicochemical properties of 139 ALDH proteins to better understand the characteristics of proteins across different ALDH families (**Figure 2**; **Supplementary Table 3**). The analysis revealed significant differences in physicochemical properties among the ALDH proteins of various ALDH families. For instance, ALDH members in family 6 showed substantial variation in both the number of amino acid residues and molecular weight, whereas those in other ALDH families exhibited relative stability. The instability indices for members of ALDH families 2, 5, 7, 11, and 18 were all below 40, indicating their relative stability. GRAVY values for members of families 12 and 18 were below 0, suggesting these proteins are hydrophilic. Moreover, there were notable differences in the two-dimensional structural compositions of proteins across different ALDH families; the average random coil proportion in family 18 members was 34.36%, whereas in other ALDH families it exceeded 41.38%. These observations provide valuable reference data for future studies on the structural and functional characteristics of ALDH proteins from different ALDH families.

### Conserved motifs and gene structural analysis

**Figure 3:**
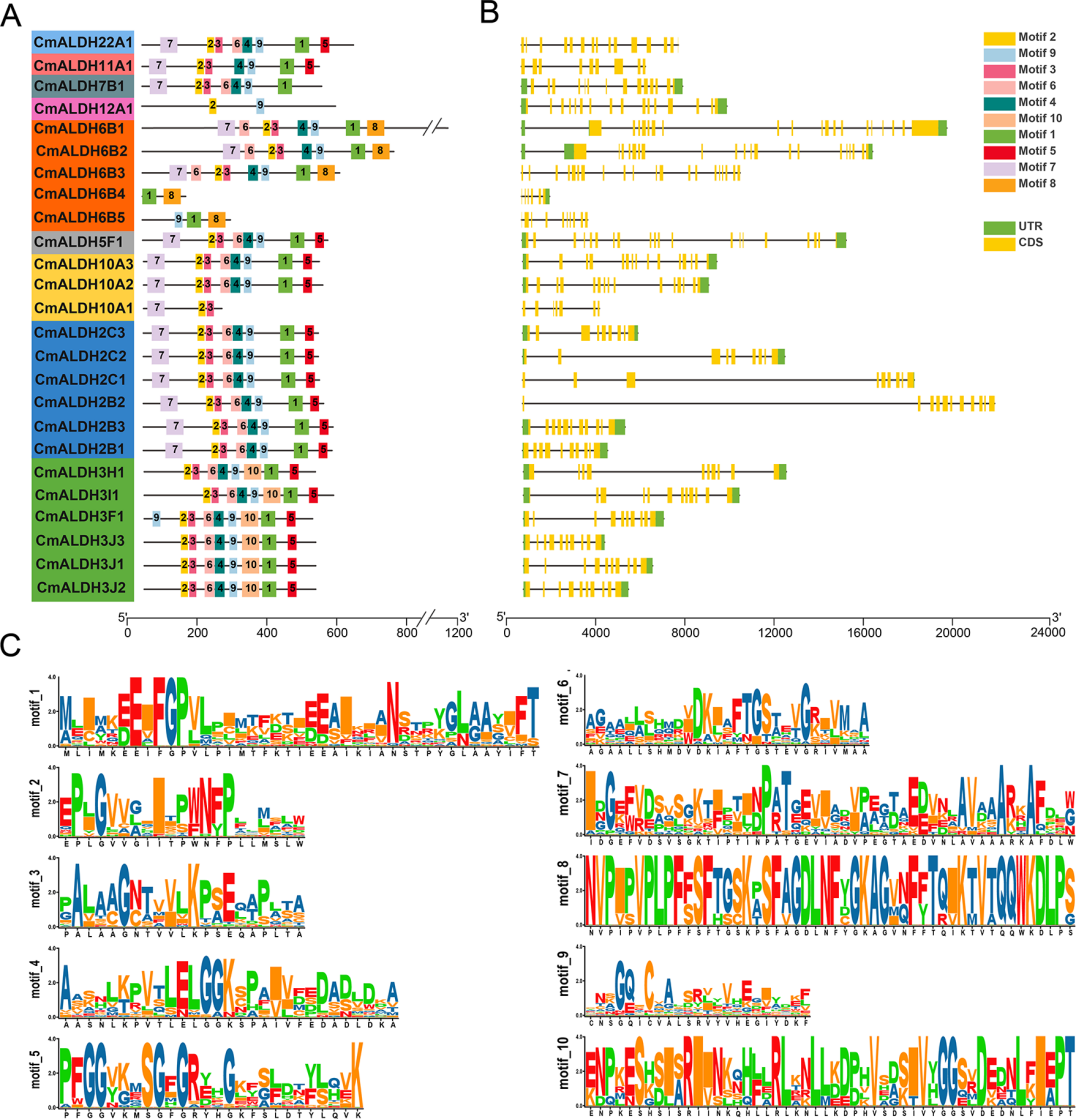
The conserved motifs and gene structure analysis of *CmALDH* genes. **(A)** The conserved motifs of *CmALDH*. **(B)** The gene structure of *CmALDH*. **(C)** The sequence of ten conserved motifs in *CmALDH* proteins.

An analysis of genetic features was conducted to elucidate the evolutionary development of *CmALDH*, as illustrated in **Figure 3**. The MEME online tool facilitated the identification of conserved motif features within CmALDH (**Figures 3A, C**). Notably, several motifs, such as Motif 1, Motif 4, and Motif 9, are widely distributed across most members of the CmALDH family. The arrangement of motifs in Family 2 members is identical, indicating strong conservation. However, certain motifs are specific to particular branches; for instance, Motif 8 is exclusively found in Family 6 members and is absent from other ALDH families. Most genes in the *CmALDH* superfamily contain between 5 and 20 introns (**Figure 3B**; **Supplementary Table 2**), a characteristic that is consistent with ALDH family members in other species such as *A. thaliana*, *O. sativa*, and *S. lycopersicum* (Islam and Ghosh, 2022).

### Chromosomal location and duplication analysis

**Figure 4:**
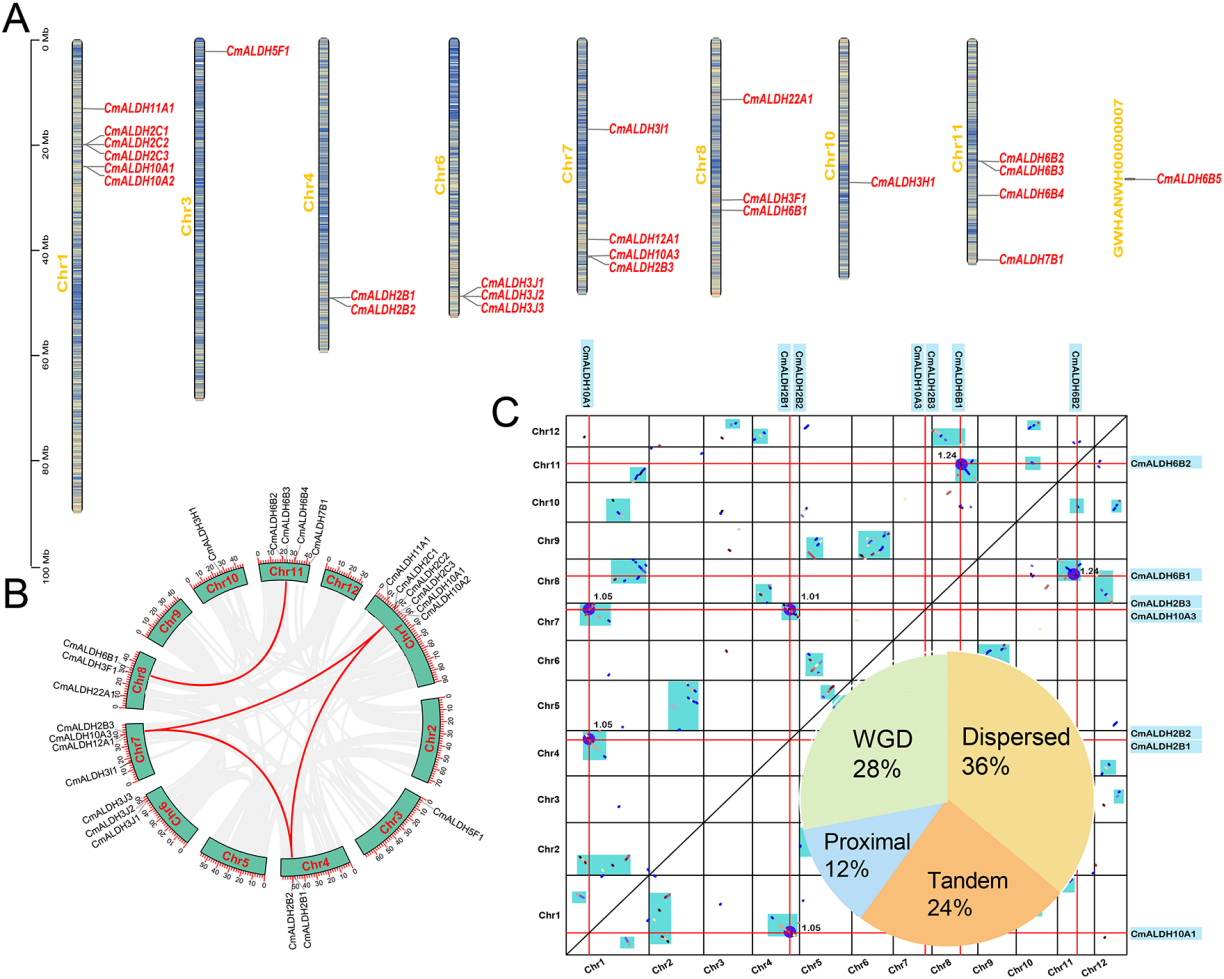
Chromosome distribution and duplication type analysis of *CmALDH* genes. **(A)** Chromosome distribution of *CmALDH* genes. The color of segments in the chromosomes shows the gene density of the corresponding region. **(B)** Collinear relationship within *C. mollissima* genome. **(C)** Homologous collinear dot-plot within *C. mollissima* genome. The collinear blocks from WGD containing *CmALDH* genes are marked in the gray boxes of the figure, and the blocks length and median Ks of the collinear blocks are marked. The genes highlighted are identified as from WGD events. Only the *CmALDH* of WGD or segmental determined by MCScanX are marked at the boundary of the dot-plot. The pie chart in the bottom right corner of the dot-plot shows the proportion of *CmALDH* for different duplication types.

Twenty-four *CmALDH* genes are distributed unevenly across eight chromosomes, with an additional gene, *CmALDH25*, located on an unanchored scaffold (**Figure 4A**). Chromosome 1 hosts the largest number of *CmALDH* members, totaling six, followed by Chromosomes 7 and 11, each of which contains four members. In contrast, Chromosomes 3 and 10 each harbor only one member. Notably, Chromosomes 1 and 6 each contain a cluster of three *CmALDH* genes, suggesting these are hotspots for gene duplication within the *CmALDH* family. Gene duplication is a critical mechanism for the expansion and functional diversification of gene families. The types of gene duplication were analyzed using the duplicate_gene_classifier tool in MCScanX (**Figure 4B**). Additionally, the Ks values of homologous genes within collinear blocks were examined, demonstrating block complementarity as previously reported (**Figure 4C**) (Yu et al., 2022a, 2025). The analysis revealed that nine *CmALDH* members originated from dispersed duplication, followed by WGD accounting for 28% (7 members), tandem duplication for 24% (6 members), and proximal duplication for 12% (3 members). These results highlight dispersed duplication as the primary mechanism driving the expansion of the CmALDH gene family. Furthermore, the Ka/Ks for homologous gene pairs was calculated, reflecting the selective pressures they have undergone (**Supplementary Table 4**). All analyzed homologous gene pairs exhibited Ka/Ks ratios below 1, suggesting that they have predominantly been subject to purifying selection throughout their evolution.

### Collinearity analysis of *ALDHs* in several plant species

**Figure 5:**
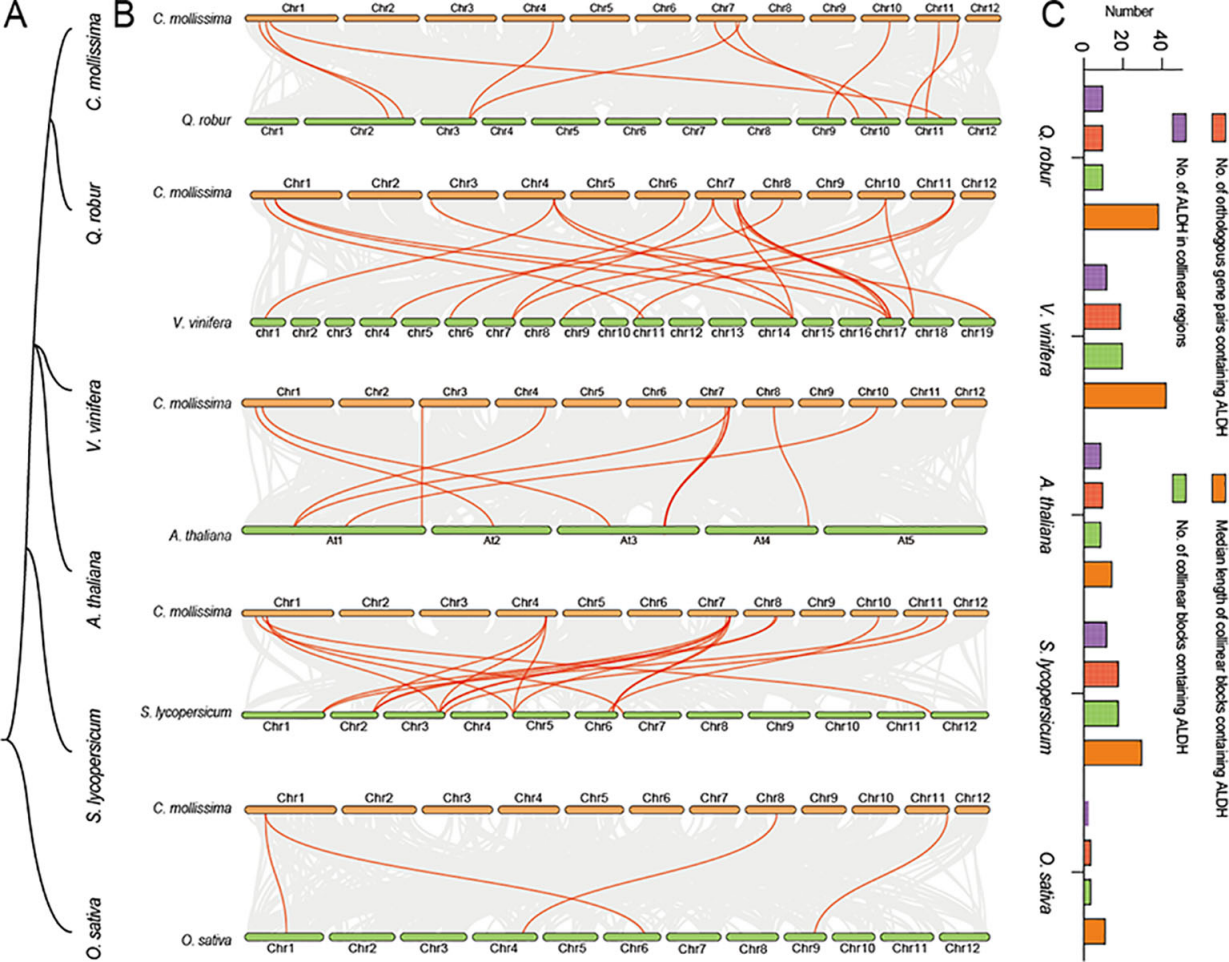
Collinear analyses between the *CmALDH* genes and genes in five representative plant species (*Q. robur*, *V. vinifera*, *A. thaliana*, *S. lycopersicum*, *O. sativa*). **(A)** The genetic relationship between six plant species. **(B)** The dual collinear plot between *C. mollissima* and five representative plant species. Gray lines in the background indicate collinear blocks within *C. mollissima* and other plant genomes, while red lines highlight collinear *ALDH* gene pairs. **(C)** The number of *CmALDH* in collinear regions, number of orthologous gene pairs containing *CmALDH*, number of collinear blocks containing *CmALDH*, and median length of collinear blocks containing *CmALDH* between *C. mollissima* and the other five plant genomes.

Gene families are often regarded as derivatives of a single ancestral gene, which expand through gene duplication events (Domazet-Lošo et al., 2024). In this study, we investigated the collinearity between *C. mollissima* and five representative plant species to elucidate the evolutionary trajectory of the CmALDH family (**Figure 5**). We identified specific numbers of *CmALDH* genes in the collinear regions between *C. mollissima* and each of the following species: *Q. robur* (10 genes), *V. vinifera* (12 genes), *A. thaliana* (9 genes), *S. lycopersicum* (12 genes), and *O. sativa* (3 genes). These genes formed 10, 19, 10, 18, and 4 orthologous pairs, respectively (**Figure 5**; **Supplementary Table 5**–**9**). Notably, certain *CmALDH* members, such as *CmALDH2B1*, established three or more orthologous pairs with species like *V. vinifera* and *S. lycopersicum*, indicating the retention of additional gene copies in these plants (**Supplementary Tables 8**, **9**). According to the gene dosage hypothesis, the presence of these additional copies suggests that these genes may play significant roles in the evolution of the ALDH superfamily or in specific metabolic pathways (Birchler and Veitia, 2007). The lengths of the collinearity blocks that contain *CmALDH* genes in *C. mollissima* and the aforementioned plants were 10, 20, 9, 18, and 4, respectively. The median lengths of these blocks were 38.3, 42.25, 14.66, 29.83, and 11.25 (**Figure 5**; **Supplementary Table 10**–**14**). Interestingly, the numerical distribution of collinear blocks containing the *CmALDH* genes in *C. mollissima* and the analyzed plants does not consistently correlate with their phylogenetic relationships. This inconsistency may be attributed to factors such as species-specific WGDs, diverse environmental pressures, and varying degrees of genome assembly completeness (Yu et al., 2022b, 2025).

### *Cis*-acting elements, and TFs regulatory analysis

**Figure 6:**
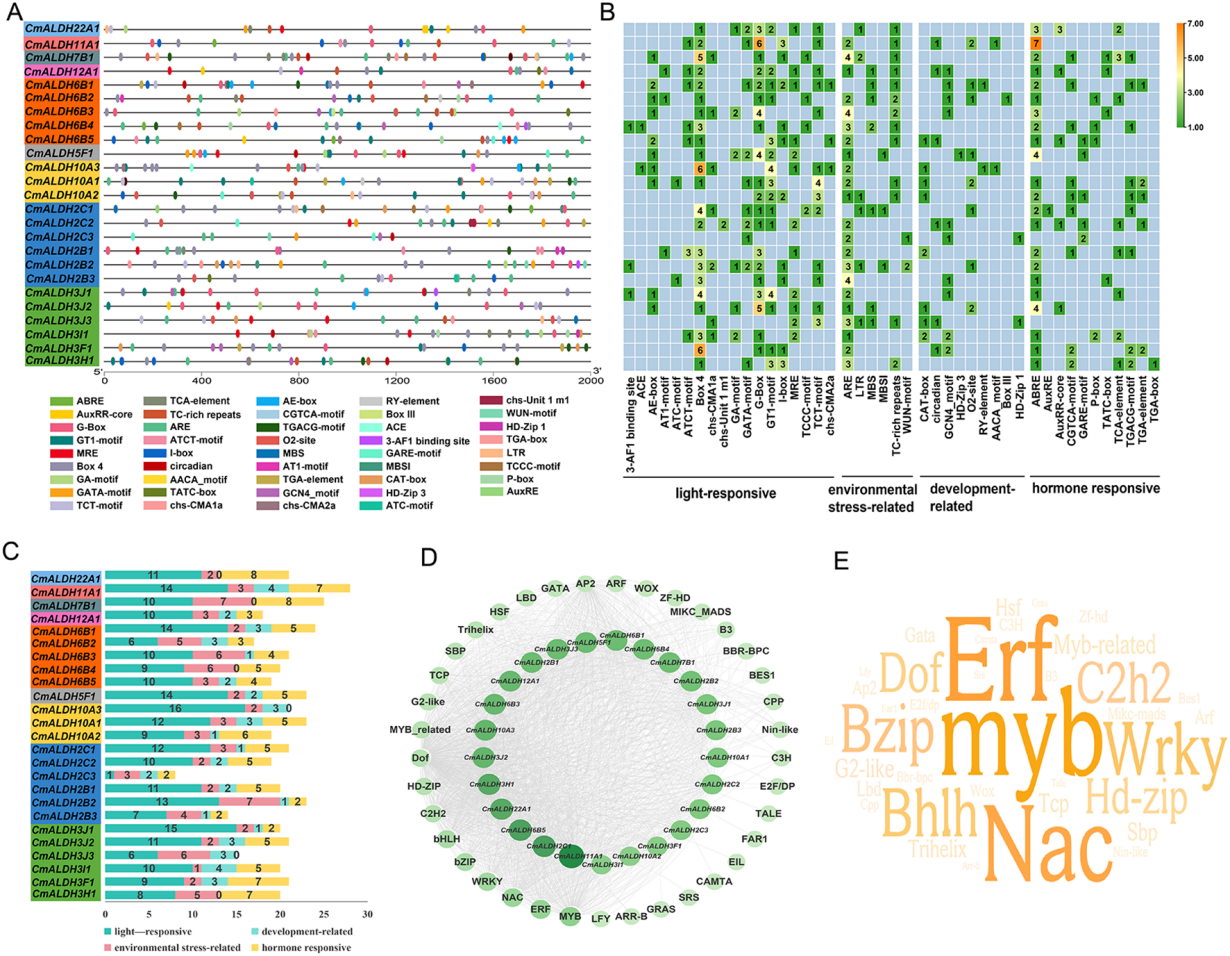
Prediction of *cis*-acting elements and transcription factor regulatory network analysis of *CmALDH*. **(A)** *Cis*-acting elements in the promoters of *CmALDH*. Various color symbols present different elements, and their position in the figure indicates their relative position on the promoter. **(B)** The number of various *cis*-acting elements in the promoters of each *CmALDH*. **(C)** The relative proportions of different *cis*-acting elements in the promoters of *CmALDH* are indicated in the chart. **(D)** The putative TF regulatory network analysis of *CmALDH*. **(E)** Word-cloud of predicted TFs interacting with *CmALDH* genes. The font size is positively correlated with the number of corresponding TFs.

To investigate the regulatory mechanisms underlying the roles of *CmALDH* genes in the growth and development of *C. mollissima* and their adaptation to environmental stresses, we analyzed the *cis*-acting elements within their promoter regions (**Figure 6A**; **Supplementary Table 15**). We identified a total of 501 *cis*-acting elements across the promoter regions of 25 *CmALDH* genes. These elements are implicated in various biological processes including light response, environmental stress, growth and development, and hormone response (**Figures 6B, C**). In terms of hormone responses, a wide distribution of elements such as abscisic acid response elements (ABRE), methyl jasmonate response elements (CGTCA-motif and TGACG-motif), auxin response elements (AuxRR-core and TGA-element), and gibberellin response elements (GARE-motif and P-box) was observed. This distribution underscores the complex regulation of *CmALDH* gene expression mediated by multiple plant hormone signals. Additionally, we identified numerous elements related to environmental stress, including MYB binding sites (MBS) for drought inducibility, TC-rich repeats for defense and stress responsiveness, and anaerobic response elements (ARE). These findings strongly indicate that *CmALDH* genes are pivotal in the response of *C. mollissima* to a variety of environmental stresses. Also, several elements associated with growth and development, as well as light responses, such as Box 4, G-Box, and GT1-motif, were identified.

To further delineate the potential transcriptional regulatory network influencing *CmALDH* genes, we predicted TFs that might interact with these genes (**Figure 6D**; **Supplementary Table 16**). The analysis revealed that 286 TFs, spanning 37 TF families, are potential regulators of the *CmALDH* genes. Notably, the families of MYB, ERF, NAC, WRKY, bZIP, and BHLH included the largest number of potential regulators (**Figure 6E**). In contrast, certain TF families, such as CAMTA, LFY, EIL, SRS, and GRAS, were represented by only a single member predicted to regulate *CmALDH*. Moreover, specific genes such as *CmALDH11A1*, *CmALDH2C1*, and *CmALDH6B5* are predicted to be regulated by as many as 90, 63, and 62 TFs, respectively. These results offer significant insights into the transcriptional mechanisms through which this gene family contributes to diverse biological processes in *C. mollissima*.

### Protein interaction network and function enrichment analysis

**Figure 7:**
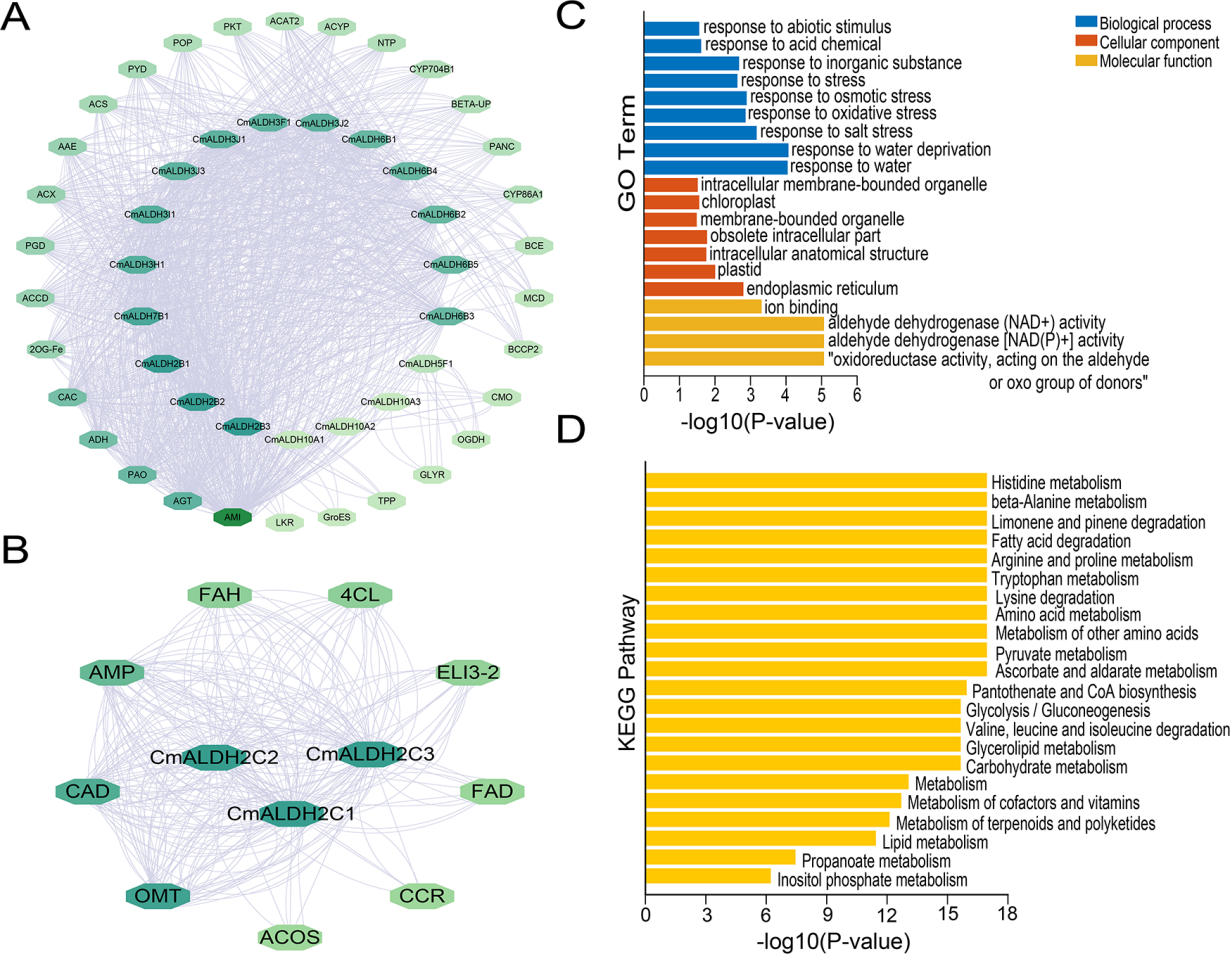
Protein interaction network and GO/KEGG analysis of *CmALDH*. **(A)** Protein interaction subnetwork 1 of CmALDH proteins. **(B)** Protein interaction subnetwork 2 of CmALDH proteins. **(C)** GO function enrichment analysis of *CmALDH* genes. **(D)** KEGG function enrichment analysis of *CmALDH* genes.

We predicted the interaction network of CmALDH proteins, which revealed that instead of forming a single interconnected cluster, the network segmented into four distinct, independent subnetworks (**Figures 7A, B**; **Supplementary Figure 4**). This observation suggests that the CmALDH superfamily might be functionally segregated into four separate subnetworks, each involved in relatively independent biological processes. Subnetwork 1, the largest, includes 19 CmALDH members, with CmALDH2B3, CmALDH2B2, and CmALDH2B1 interacting with the highest number of other proteins (**Figure 7A**). Subnetwork 2 comprises only three CmALDH members: CmALDH2C1, CmALDH2C2, and CmALDH2C3 (**Figure 7B**). These results indicate that members of family 2 are central to the constitution of the CmALDH protein interaction network and likely play important roles in the biological functions of this family. The other two subnetworks contain only one CmALDH member each: CmALDH11A1 and CmALDH12A1, respectively (**Supplementary Figure 4**). Additionally, we described the potential functions of all *CmALDH* genes using annotation information from the GO and KEGG databases (**Figures 7C, D**). GO functional enrichment analysis showed that *CmALDHs* were significantly enriched in biological processes such as “response to abiotic stimulus,” “response to acid chemical,” “response to stress,” and “response to salt stress,” underscoring their important roles in *C. mollissima* responses to environmental stresses (**Figure 7C**). KEGG functional enrichment analysis indicated that *CmALDHs* were highly enriched in metabolic pathways, including “histidine metabolism,” “beta-alanine metabolism,” and “limonene and pinene degradation.” These pathways are involved in the production of reactive aldehyde intermediates, which are established substrates for ALDH enzymes (**Figure 7D**). These findings align with evidence from other studies suggesting that *ALDH* genes are involved in signal transduction and responses to biotic and abiotic stresses (Kirch et al., 2004; Li et al., 2023; Xu et al., 2023; Yang et al., 2025).

### Expression pattern analysis of *CmALDH* genes under abiotic stress

**Figure 8:**
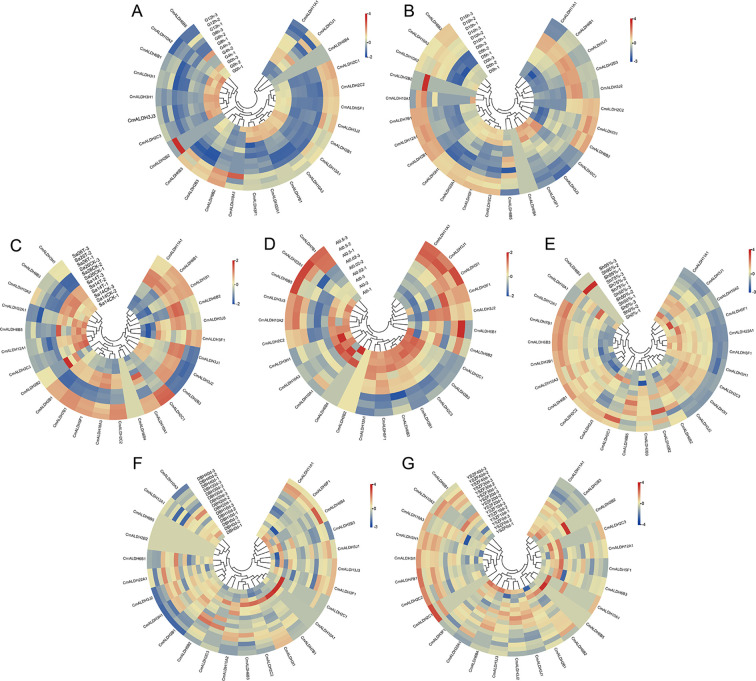
Expression profiles of *CmALDH* genes under different abiotic stresses. **(A)** The expression profiles of *CmALDH* genes under high−temperature stress (45 °C) at various time points. G0h, G4h, G8h, and G12h: 0, 4, 8, and 12 h of treatment. **(B)** The expression profiles of *CmALDH* genes under low−temperature stress (−15 °C) at various time points. D0h, D5h, D10h, and D15h: 0, 5, 10, and 15 h of treatment. **(C)** The expression profiles of *CmALDH* genes of ‘Yanshanzaofeng’ after 14 and 26 days of salt stress treatment (200 mmol/L NaCl). 14CK, 14T: control and treatment at 14 d; 26CK, 26T: control and treatment at 26 d. **(D)** The expression profiles of *CmALDH* genes under different levels of alkaline stress (0.02 g/L and 0.5 g/L Na_2_CO_3_ for 7 d). CK: control; T0.02: 0.02 g/L Na_2_CO_3_; T0.5: 0.5 g/L Na_2_CO_3_. **(E)** The expression profiles of *CmALDH* genes in leaves under 0 %, 50 %, 75 %, and 95 % shade. **(F)** The expression profiles of *CmALDH* genes in leaves of cultivar ‘Dabanhong’ treated with drought for 0, 10, 20, 30, and 40 days. **(G)** The expression profiles of *CmALDH* genes in leaves of cultivar ‘Yanshanzaofeng’ treated with drought for 0, 10, 20, 30, and 40 days. The expression profiles shown in **(A–G)** are derived from transcriptome data.

**Figure 9:**
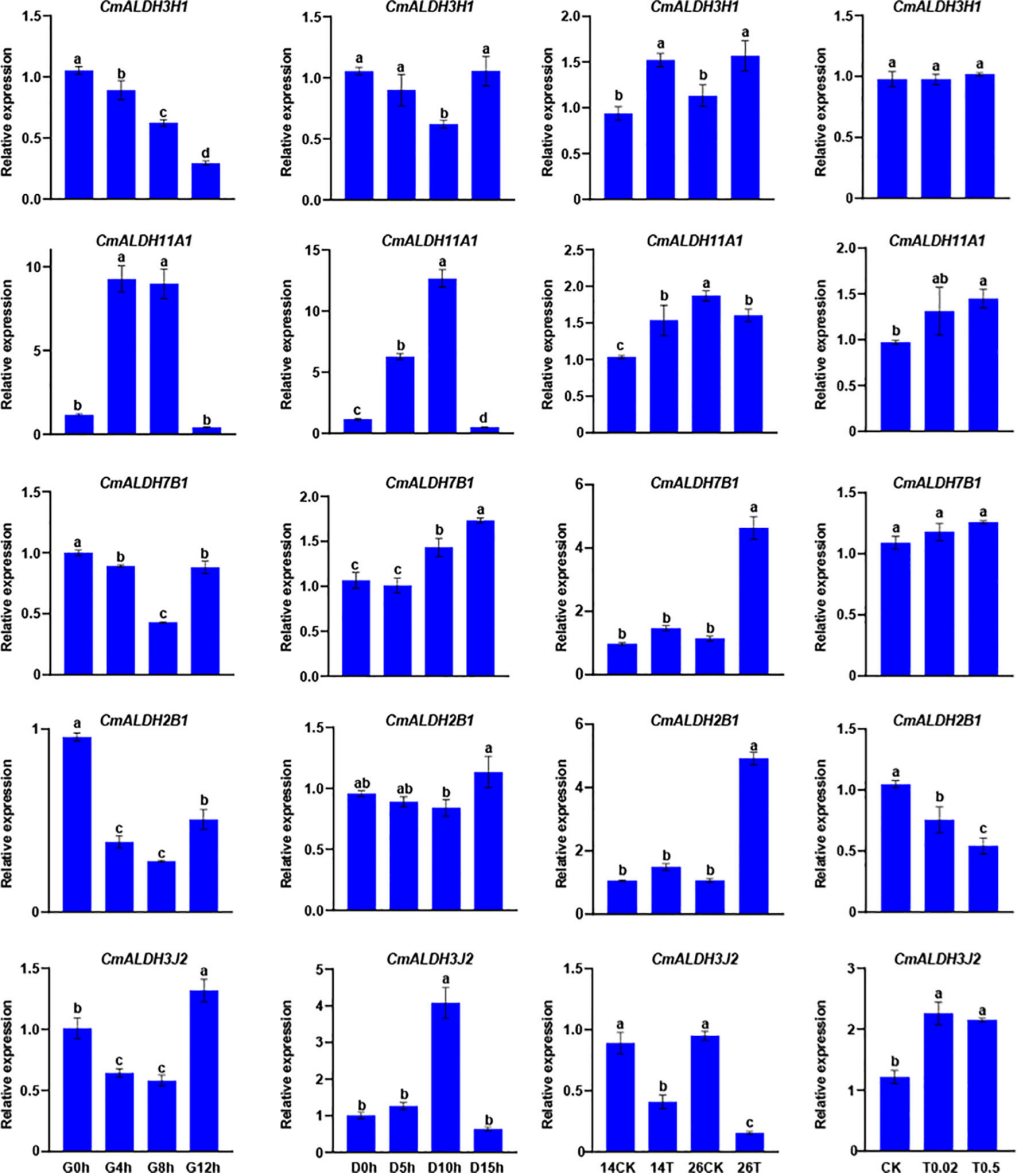
RT−qPCR validation of *CmALDH* genes expression in leaves of *C. mollissima* ‘Yanshanzaofeng’ under abiotic stresses. The expression levels of five selected *CmALDH* genes (*CmALDH3H1*, *CmALDH11A1*, *CmALDH7B1*, *CmALDH2B1*, and *CmALDH3J2*) were examined under high−temperature stress (45 °C; G0h, G4h, G8h, and G12h: 0, 4, 8, and 12 h), low−temperature stress (−15 °C; D0h, D5h, D10h, and D15h: 0, 5, 10, and 15 h), salt stress (200 mmol/L NaCl; 14CK, 14T: control and treatment at 14 d; 26CK, 26T: control and treatment at 26 d), and alkaline stress (0.02 g/L and 0.5 g/L Na_2_CO_3_ for 7 d; CK: control, T0.02: 0.02 g/L, T0.5: 0.5 g/L). Lowercase letter(s) above the bars indicate significant differences (α = 0.05, LSD) among the treatments.

The expression profiles of *CmALDH* genes were analyzed under various abiotic stresses including high temperature, low temperature, drought, salt stress, alkaline stress, and shading to explore their potential roles in the responses of *C. mollissima* to these conditions (**Figure 8**). Under high temperature stress, significant changes in the expression levels of several genes were observed (**Figure 8A**). For instance, the FPKM values of *CmALDH3H1* decreased to 27.52%, 21.21%, and 26.16% of the control levels at 4, 8, and 12 hours of stress, respectively, demonstrating a substantial impact on its expression. Conversely, low temperature stress significantly induced the expression of multiple *CmALDH* genes, such as *CmALDH11A1* and *CmALDH2C2*. Specifically, compared to the control (FPKM: 103.79), the expression of *CmALDH11A1* was elevated by 200% (FPKM: 323.56) and 500% (FPKM: 667.13) after 5 and 10 hours of exposure to low temperatures, respectively (**Figure 8B**). Salt stress also led to a notable upregulation of several genes, including *CmALDH2C2*, *CmALDH2B1*, and *CmALDH7B1*. For example, the FPKM values of *CmALDH2C2* increased from 46.76 in the control to 98.93 and from 51.50 to 130.74 after 14 and 26 days of salt stress, respectively. In contrast, the expression levels of certain genes, such as *CmALDH11A1*, *CmALDH3J1*, *CmALDH3J2*, and *CmALDH2B3*, were downregulated as salt stress persisted (**Figure 8C**). During alkaline stress treatment, *CmALDH11A1* showed upregulation, while *CmALDH2C1*, *CmALDH2B1*, and *CmALDH6B3* experienced significant downregulation compared to the control (**Figure 8D**). Based on transcriptome analysis, genes including *CmALDH3I1*, *CmALDH10A3*, and *CmALDH2B1* were significantly upregulated, whereas *CmALDH3J1*, *CmALDH3J2*, *CmALDH22A1*, and *CmALDH3F1* exhibited marked downregulation under shading stress (**Figure 8E**). Furthermore, under various levels of drought stress, *CmALDH3I1* and *CmALDH7B1* were significantly upregulated in both ‘Dabanhong’ and ‘Yanshanzaofeng’ cultivars of *C. mollissima* according to transcriptome data, suggesting their potential roles in the species’ response to drought stress (**Figures 8F, G**). RT-qPCR experiments validated the expression levels of *CmALDH* genes under high temperature, low temperature, salt, and alkaline stresses (**Figure 9**). The results generally corresponded with those of the transcriptome analysis, thereby confirming the reliability of the transcriptome data. The expression patterns under drought and shading stresses are derived from transcriptome data only and await further experimental validation.

## Discussion

The ALDH superfamily plays crucial roles in plant development and in responding to stress. This study systematically characterized the ALDH superfamily in the *C. mollissima* genome, identifying 25 *CmALDH* genes distributed across nine known families (**Figure 1**). The number of identified genes is comparable to that in *V. vinifera* (23 genes), but significantly lower than in *Phyllostachys edulis* with 60 genes and *Arachis hypogaea* with 71 genes, both of which have undergone recent WGD events (Zhang et al., 2012; Xu et al., 2023; Zhang et al., 2023). These findings suggest that the expansion of the ALDH superfamily in different plant lineages correlates with their unique genomic evolutionary histories, particularly WGD events. Phylogenetic and gene structure analyses revealed highly conserved motif compositions and gene structures within the same family, while notable differences were observed between families (**Figure 3**), potentially underpinning the functional diversity within the ALDH superfamily (Kirch et al., 2004).

Gene duplication is acknowledged as a primary driver of gene family evolution and functional innovation (Qiao et al., 2019). Analysis of duplication types indicated that dispersed duplication was the predominant mechanism facilitating the expansion of the CmALDH superfamily (**Figure 4B**). Dispersed duplication has been instrumental in the expansion of numerous plant gene families (Liu et al., 2025; Yu et al., 2025b). Collinearity analysis identified several ALDH collinear homolog pairs between *C. mollissima* and its closely related species *Q. robur* and *V. vinifera* (**Figure 5**). This reflects genomic conservation within the Fagaceae and Vitaceae families, which has contributed to genomic stability throughout their evolution. Notably, genes such as *CmALDH2B1* exhibit collinearity with multiple distantly related species, such as *S. lycopersicum* and *A. thaliana*, suggesting that these genes likely originated from ancient duplication events and retain essential, conserved functions across diverse plant lineages (Freeling, 2009).

Analysis of the *CmALDH* genes’ promoters revealed an abundance of elements associated with responses to abscisic acid (ABRE), methyl jasmonate (CGTCA/TGACG-motif), and auxin (AuxRR-core/TGA-element) (**Figures 6A–C**). This suggests that *CmALDH* expression is regulated by multiple plant hormone signaling pathways, which may be vital for integrating environmental stress and developmental responses in plants (Verma et al., 2016). Additionally, numerous elements associated with drought (MBS), cold, defense (TC-rich repeats), and anaerobic (ARE) stress were identified, implying the extensive involvement of *CmALDHs* in responding to abiotic stresses. Further prediction of TF regulatory networks revealed that core stress response TF families, including MYB, ERF, NAC, and WRKY, are crucial in regulating CmALDH (**Figures 6D, E**) (Nakashima et al., 2012). GO and KEGG enrichment analyses (**Figures 7C, D**) further connected *CmALDH* to biological processes such as “response to abiotic stress” and “response to salt stress,” along with metabolic pathways involving active aldehyde intermediates, including histidine metabolism and β-alanine metabolism. This underscores the role of *CmALDH* in the stress adaptation of *C. mollissima* (Kotchoni et al., 2006).

Transcriptomic analysis revealed that multiple *CmALDH* genes exhibit differential expression under all six abiotic stresses tested (high temperature, low temperature, drought, salt, alkalinity, and shading). RT−qPCR validation (performed for high temperature, low temperature, salt, and alkaline stresses) consistently confirmed these expression patterns for the validated conditions (**Figure 8** and **9**). Notably, distinct expression patterns were observed among different ALDH family members, suggesting functional specialization within the ALDH superfamily in response to specific stress conditions. For instance, *CmALDH11A1* was strongly induced by low−temperature, salt, and alkaline stresses. In *A. thaliana*, *AtALDH11A1* has been shown to participate in aldehyde detoxification under oxidative stress conditions, and its expression is responsive to multiple abiotic stimuli (Kotchoni et al., 2006; Missihoun et al., 2018). Similarly, *CmALDH2C2* was significantly upregulated under both low−temperature and salt stresses. Homologous *ALDH2* genes in *O. sativa* and *Z. mays* have been implicated in stress tolerance by reducing aldehyde toxicity and maintaining redox balance (Gao and Han, 2009; Du et al., 2022). *CmALDH2B1* exhibited upregulation under salt, alkaline, and shading stresses, suggesting a broader role in mediating cross−tolerance to multiple environmental constraints—a pattern also observed for *ALDH2*B homologs in *Sorghum bicolor* and *C. annuum* (Islam et al., 2022; Yang et al., 2025). Under drought and shading stresses, *CmALDH3I1* was induced, while *CmALDH3J1* responded to salt and shading conditions. Members of the ALDH3 family have been functionally characterized in various plant species. In these species, they contribute to stress tolerance by scavenging reactive aldehydes generated during lipid peroxidation (Kirch et al., 2004; Xu et al., 2023). Notably, based on transcriptome data, *CmALDH3I1* and *CmALDH7B1* were consistently upregulated under drought stress in two distinct *C. mollissima* cultivars, ‘Yanshanzaofeng’ and ‘Dabanhong’, suggesting that they may play important roles in the drought stress response of *C. mollissima*. In *A. hypogaea*, AhALDH7 family members have been reported to respond to saline−alkali stress, supporting the potential involvement of *ALDH7* genes in abiotic stress adaptation (Zhang et al., 2023). The expression patterns of these *CmALDH* genes are consistent with the presence of diverse stress−related cis−acting elements in their promoter regions, including ABRE (abscisic acid responsiveness), MBS (drought inducibility), and TC−rich repeats (defense and stress responsiveness) (**Figure 6**). These regulatory elements likely enable rapid transcriptional reprogramming under adverse conditions, allowing *CmALDH* genes to integrate multiple stress signals and contribute to the overall stress resilience of *C. mollissima*. Collectively, these findings suggest potential roles of *CmALDH* genes in the abiotic stress responses of *C. mollissima*. The observed expression diversity reflects both functional conservation and neo−functionalization within the ALDH superfamily, providing a valuable foundation for future functional validation and genetic improvement of stress tolerance in *C. mollissima*.

In summary, this study systematically elucidates the characteristics of the ALDH superfamily in *C. mollissima*, their evolutionary dynamics, and their potential functions in abiotic stress responses. The RT−qPCR validation conducted for high temperature, low temperature, salt, and alkaline stresses confirms the reliability of the transcriptome data, while the expression patterns under drought and shading stresses provide valuable candidates for future functional characterization. Further studies, including genetic transformation or mutant analysis, will be instrumental in elucidating the precise roles of individual *CmALDH* genes and advancing genetic improvement of stress tolerance in *C. mollissima*.

## Data Availability

The original contributions presented in the study are included in the article/**Supplementary Material**, further inquiries can be directed to the corresponding author/s.

## Supplementary material

Supplementary Figure 1 RNA electropherograms from *C. mollissima* leaves used for RT−qPCR analysis.

Supplementary Figure 2 Schematic diagram of the experimental design for RT−qPCR validation of *CmALDH* genes expression under abiotic stress treatments.

Supplementary Figure 3 The predicted three-dimensional structure of CmALDH protein.

Supplementary Figure 4 Protein interaction subnetwork 3 and 4 of CmALDH proteins.

Supplementary Table 1 Primers used for RT-qPCR analysis

Supplementary Table 2 Accession numbers and characteristics of 25 *CmALDH* genes in *Castanea mollissima*.

Supplementary Table 3 The physicochemical properties and two-dimensional structure prediction of members of the ALDH superfamily (from six plant species: *C. mollissima*, *A. thaliana*, *O. sativa*, *S. bicolor*, *V. vinifera*, *Z. mays*) among ten families.

Supplementary Table 4 The duplication model and calculation of Ka and Ks ratios of duplicated *CmALDH* gene pairs.

Supplementary Table 5 The orthologous gene pairs containing *CmALDH* in the cross of *Castanea mollissima* and *A. thaliana*.

Supplementary Table 6 The orthologous gene pairs containing *CmALDH* in the cross of *Castanea mollissima* and *Q. robur*.

Supplementary Table 7 The orthologous gene pairs containing *CmALDH* in the cross of *Castanea mollissima* and *V. vinifera*.

Supplementary Table 8 The orthologous gene pairs containing *CmALDH* in the cross of *Castanea mollissima* and *S. lycopersicum*.

Supplementary Table 9 The orthologous gene pairs containing *CmALDH* in the cross of *Castanea mollissima* and *O. sativa*.

Supplementary Table 10 The collinear blocks containing *CmALDH* identified between *Castanea mollissima* and *A. thaliana*.

Supplementary Table 11 The collinear blocks containing *CmALDH* identified between Castanea mollissima and *Q. robur*.

Supplementary Table 12 The collinear blocks containing *CmALDH* identified between Castanea mollissima and *V. vinifera*.

Supplementary Table 13 The collinear blocks containing *CmALDH* identified between *Castanea mollissima* and *S. lycopersicum*.

Supplementary Table 14 The collinear blocks containing *CmALDH* identified between Castanea mollissima and *O. sativa*.

Supplementary Table 15 Information of *cis*-acting elements predicted from 2000 bp upstream regions of 25 *CmALDH* genes.

Supplementary Table 16 Predictive transcription factor (TFs) analysis of *CmALDH* genes
