# Supplementary figures and images for: Genome-wide analysis of the ALDH superfamily in Castanea mollissima highlights roles in abiotic stress responses
Source: Front Plant Sci. 2026 Jun 2;17:1808001. doi: 10.3389/fpls.2026.1808001 (PMC13273040; doi:10.3389/fpls.2026.1808001)

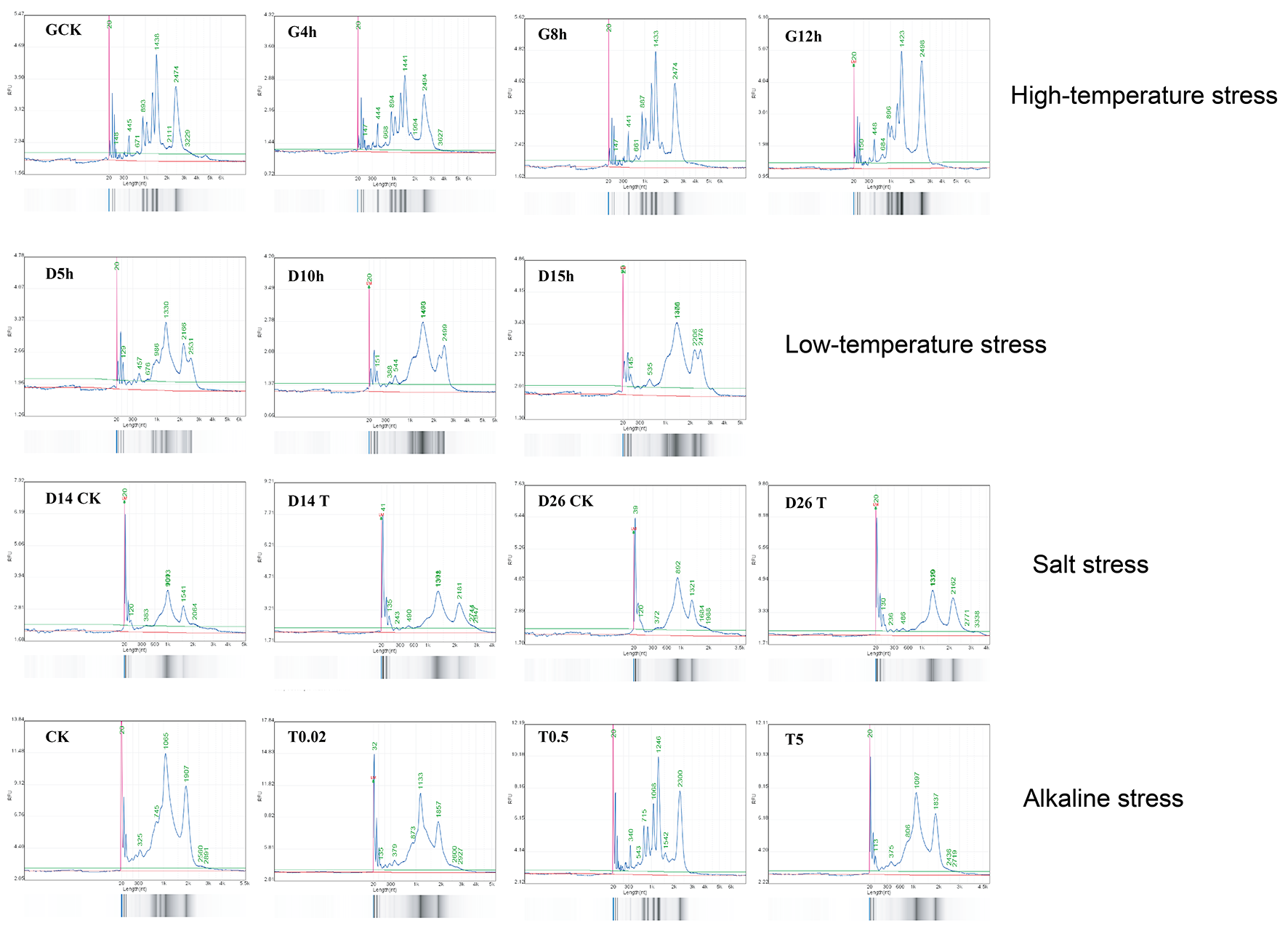

Supplement: Supplementary Figure 1 — RNA electropherograms from C. mollissima leaves used for RT−qPCR analysis. [file Image1.tif]

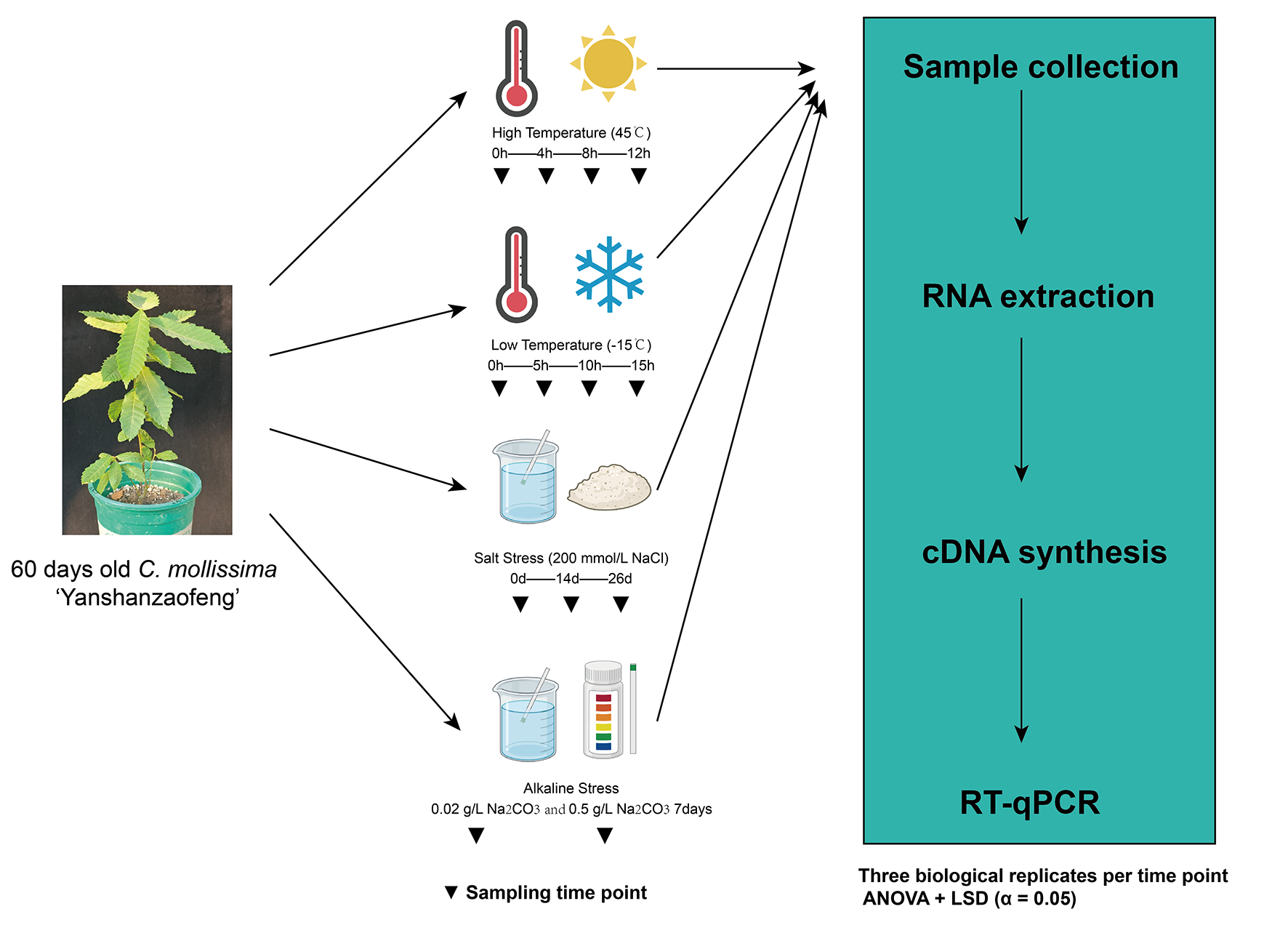

Supplement: Supplementary Figure 2 — Schematic diagram of the experimental design for RT−qPCR validation of CmALDH genes expression under abiotic stress treatments. [file Image2.tif]

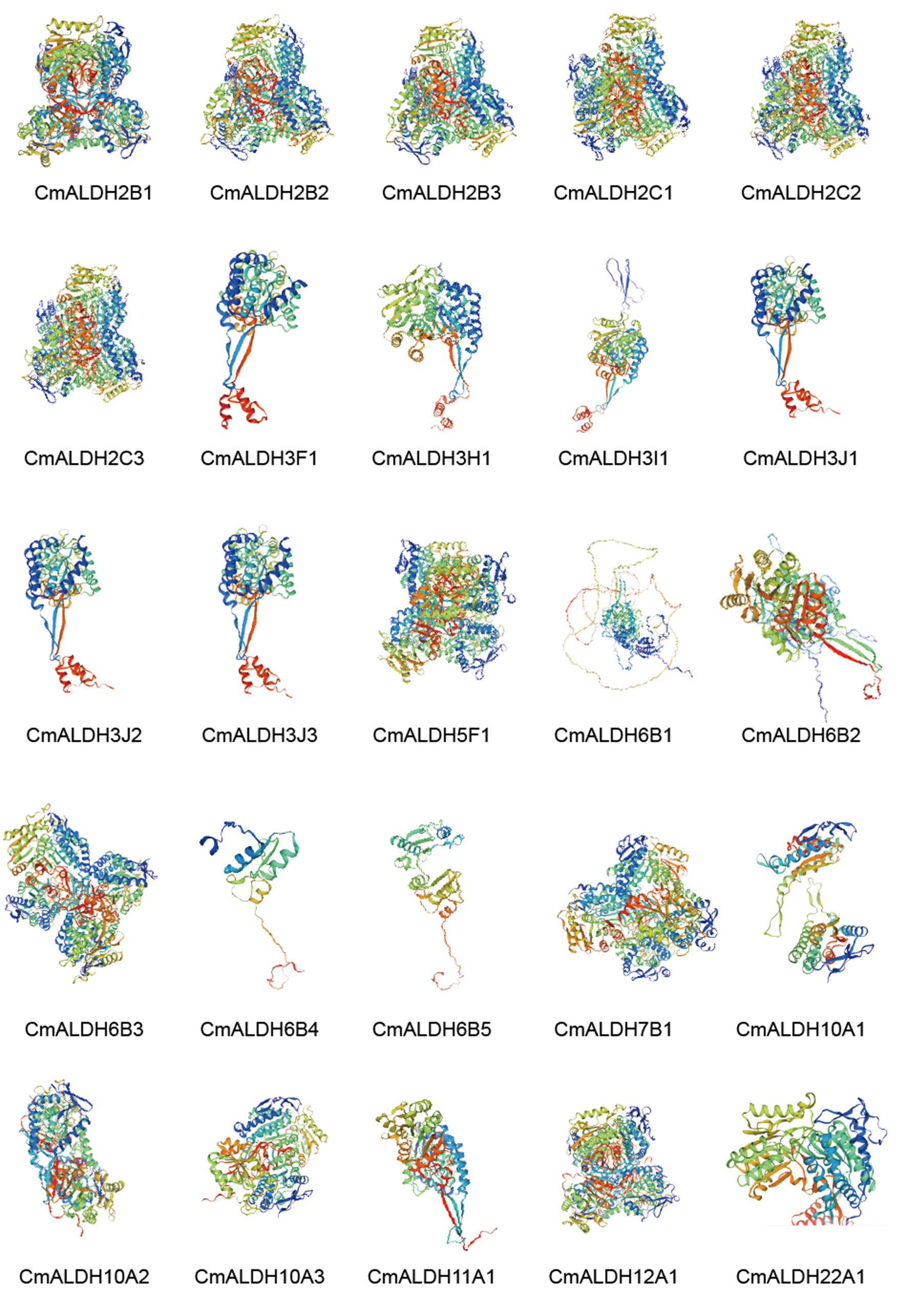

Supplement: Supplementary Figure 3 — The predicted three-dimensional structure of CmALDH protein. [file Image3.tif]

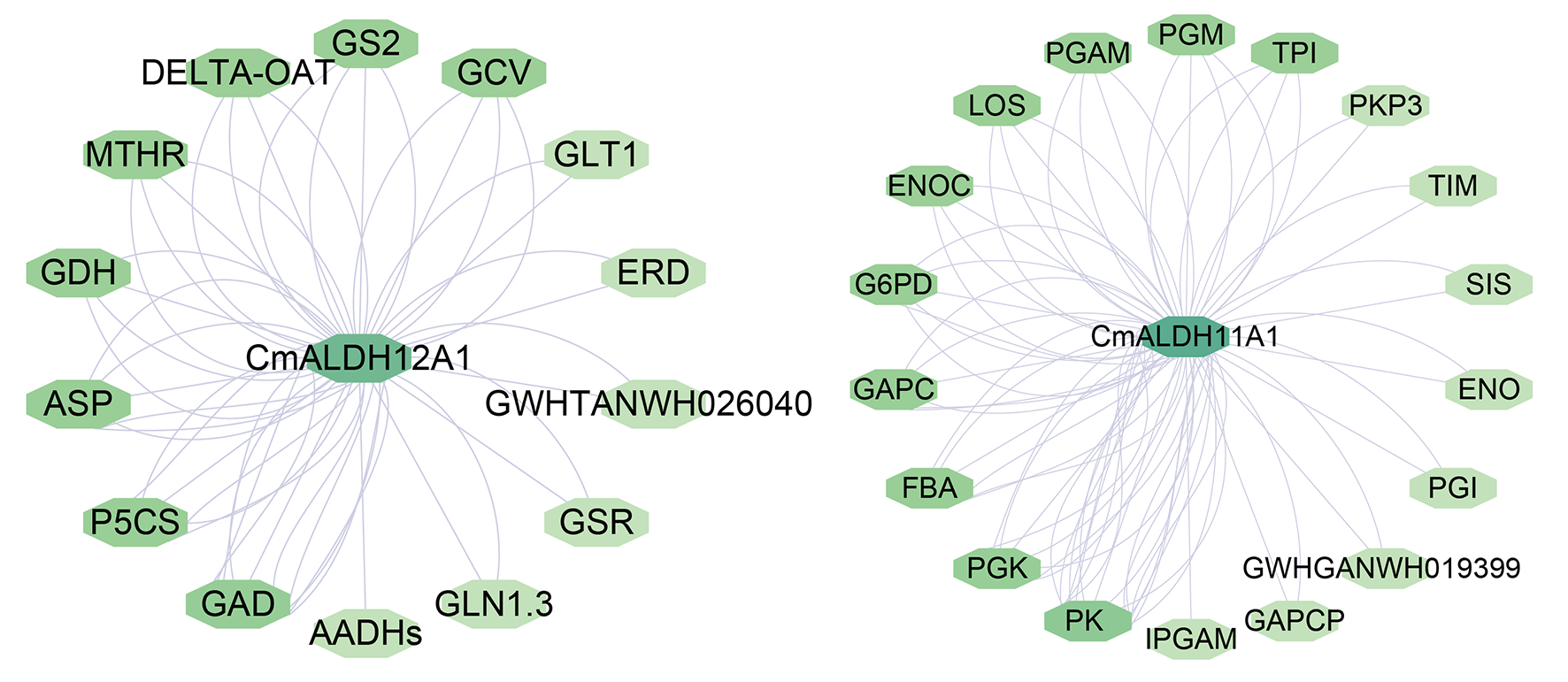

Supplement: Supplementary Figure 4 — Protein interaction subnetwork 3 and 4 of CmALDH proteins. [file Image4.tif]
